# Alkali-metal bases in catalytic hydrogen isotope exchange processes

**DOI:** 10.1039/d3cy00825h

**Published:** 2023-07-18

**Authors:** Andreu Tortajada, Eva Hevia

**Affiliations:** a Department für Chemie und Biochemie, Universität Bern Freiestrasse 3 3012 Bern Switzerland andreu.tortajadanavarro@unibe.ch eva.hevia@unibe.ch

## Abstract

The preparation of compounds labelled with deuterium or tritium has become an essential tool in a range of research fields. Hydrogen isotope exchange (HIE) offers direct access to said compounds, introducing these isotopes in a late stage. Even though the field has rapidly advanced with the use of transition metal catalysis, alkali-metal bases, used as catalysts or under stoichiometric conditions, have also emerged as a viable alternative. In this minireview we describe the latest advances in the use of alkali-metal bases in HIE processes, showcasing their synthetic potential as well as current challenges in the field. It is divided in different sections based on the isotope source used, emphasizing their benefits, disadvantages and limitations. The influence on the choice of alkali-metal in these processes as well as their possible mechanistic pathways are also discussed.

## Abstract 2

Alkali-metal bases can also catalyse Hydrogen Isotope Exchange reactions. This minireview highlights the last developments of the field, their benefits and limitations.

## Graphical abstract

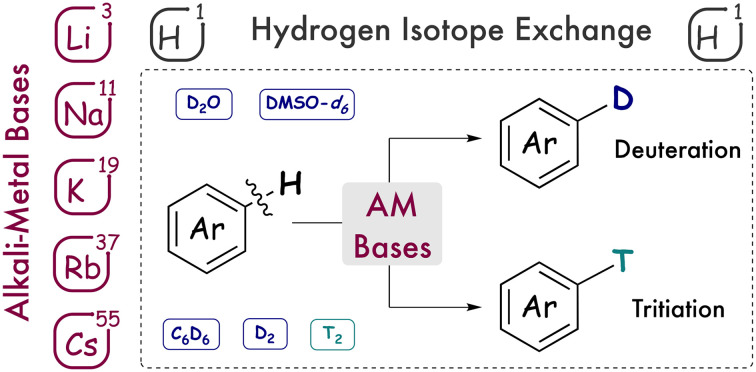

## Introduction

**Fig. 1:**
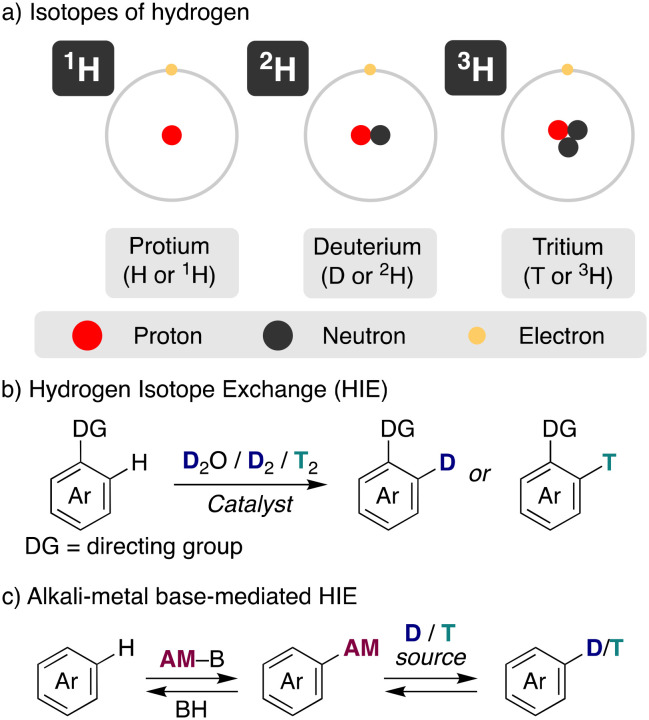
General overview of hydrogen isotope exchange (HIE) reaction. a) isotopes of hydrogen, b) hydrogen isotope exchange, c) alkali-metal base-mediated HIE.

Compounds with labelled positions with different hydrogen isotopes, deuterium or tritium (Fig. 1a), have become valuable tools in a range of research fields. For example, they are essential in supporting drug discovery and development in medicinal chemistry, obtaining materials with improved properties or allowing the investigation of chemical reaction pathways.^1^ Deuterium is a stable isotope of hydrogen, with an atomic mass of 2.014 u, a 2-fold increase with respect to protium, which confers distinctive properties to the deuterated compounds. On the other side, tritium is a radioisotope that undergoes a β-decay leading to a ^3^He and features a specific activity of 9700 Ci per g. Because of the different properties of isotopically labelled compounds (*i.e.* D_2_O and T_2_O have higher density and higher melting and boiling points than H_2_O) and their use on different applications, development of methodologies for the preparation of compounds incorporating deuterium and tritium have attracted a continued attention during the years, especially after the seminal developments of heterogeneous catalysis for isotopic incorporation in the 1960's.^2^

The traditional approach to incorporate these isotopes into organic molecules has been the use of deuterated/tritiated synthons, which are limited and require in some cases the redesign of the synthetic route. An alternative straightforward access is hydrogen isotope exchange (HIE), where the introduction of deuterium or tritium is performed in a late stage, rendering exchange of hydrogen for its isotopes *via* a C–H activation pathway (Fig. 1b).^3^ This is the preferred way, for example, in the synthesis of deuterated drugs or the preparation of stable-isotope-labelled internal standards (SILS) for quantification studies using LC–MS. However, this more direct preparation of the isotope-labelled molecules with a considerable reduced number of synthetic steps comes often at the cost of regioselectivity and chemoselectivity. Despite the challenges, in recent years important developments have been achieved in this field, allowing the specific incorporation of the desired isotope with incorporations higher than 90%. The main efforts have been focused on transition-metal catalysed HIE processes, often using arenes with directing groups as substrates and deuterium or tritium gas as the isotope source.^2^ Iridium^4^ and other noble transition metals^5^ have excelled in this task, allowing the HIE under mild conditions (low temperatures, short reaction times and no additives).^6^

In addition to transition metals, acid or base-catalysis can be used as well for HIE processes.^7^ They use generally cheaper and less complex reagents, representing a simpler and more accessible way for the incorporation of deuterium and tritium into organic molecules, with sometimes complementary selectivity to transition metals, making them an attractive pathway. In this case, the main isotopic sources used have been heavy water (D_2_O), DMSO-*d*_6_, C_6_D_6_, *i*PrOH-*d*_8_ or MeOH-*d*_4_, always in a large excess as the solvent of the reaction to allow good deuterium incorporations. However, harsher reaction conditions were required traditionally with acids or bases to achieve the incorporation of the desired isotopes *via* HIE, reducing the tolerance to more fragile functional groups. Furthermore, base-catalysed methods have shown little promise to promote HIE in substrates that contain weakly activated protons (in terms of p*K*_a_ values). In this minireview we focus our attention on the use of alkali-metal bases for hydrogen-isotope exchange (Fig. 1c), showing recent advances of these main-group reagents in these catalytic transformations, analysing the mechanistic pathways of these reactions and the effect of the nature of the alkali-metal. Moreover, we aim to highlight the synthetic potential of this approach as well as current challenges that are still needed to be solved.

## Hydrogen isotope exchange with alkali-metal bases

Hydrogen isotope exchange reactions are equilibrium processes, where high isotopic incorporations are difficult to achieve with stoichiometric amounts of the isotopic enriched source. Therefore, they are usually performed in the presence of an excess of the labelling agent, being often the solvent of the reaction. Although these processes have been known for decades, in recent years this area has attracted a renewed interest pushed by the particular properties of the resulting isotopically labelled compounds. Driven by the search of more sustainable processes, alternatives to precious transition metals and the use of different isotope sources have been reported recently. In this review, focused on the use of alkali-metal bases in HIE, we have divided the processes according to the isotopic source used, going from heavy water or deuterated DMSO to the less polar deuterated benzene, D_2_ or T_2_.

### HIE using supercritical D_2_O

**Fig. 2:**
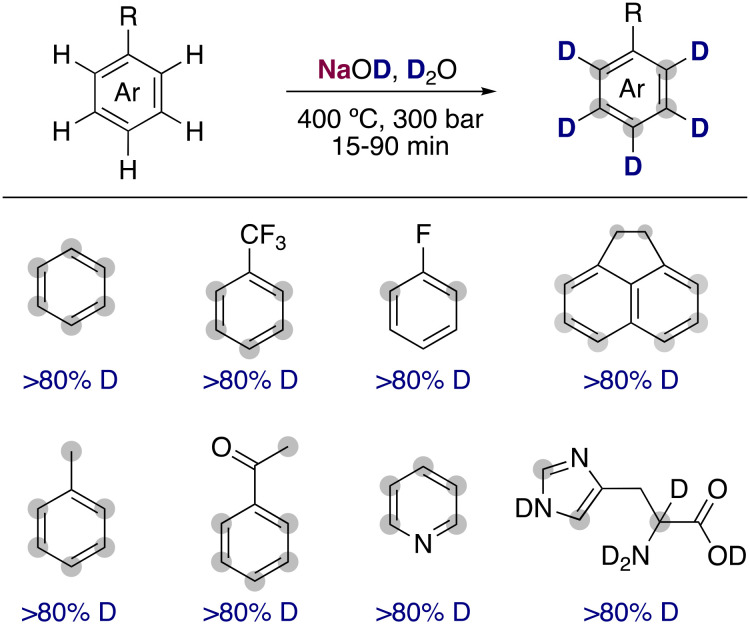
Hydrogen isotope exchange with supercritical D_2_O and NaOD as a catalyst.

The use of water upon exceeding its critical point leads to a dramatic difference of the properties of the dissolved organic molecules present. The large decrease in the dielectric and self-ionization constant of deuterium oxide in conjunction with the high temperatures can lead to a large increase of the rate constants for the reaction of very weak acidic organic molecules in hydrogen isotope exchanges. Evilia and co-workers estimated that the p*K*_a_ of benzene under supercritical conditions (400 °C and 300 bar) is reduced from 43 (room temperature) to 19.^8^ Under these conditions, they found that the simple alkali-metal base NaOD at low concentrations was able to efficiently allow the deuterium incorporation in very unactivated arenes such as benzene, toluene or 1,1,1-trifluorotoluene (Fig. 2). Shortly after, the use of supercritical conditions was extended into the deuteration of amino acids and other heterocycles, valuable non-radioactive isotopic tracers in biological systems.^9^ However, the drastic conditions promoted the racemization of the amino acids and the decomposition of some more functionalized substrates, limiting the broader applicability of this transformation.

### DMSO-*d*_6_ as deuterium source

**Fig. 3:**
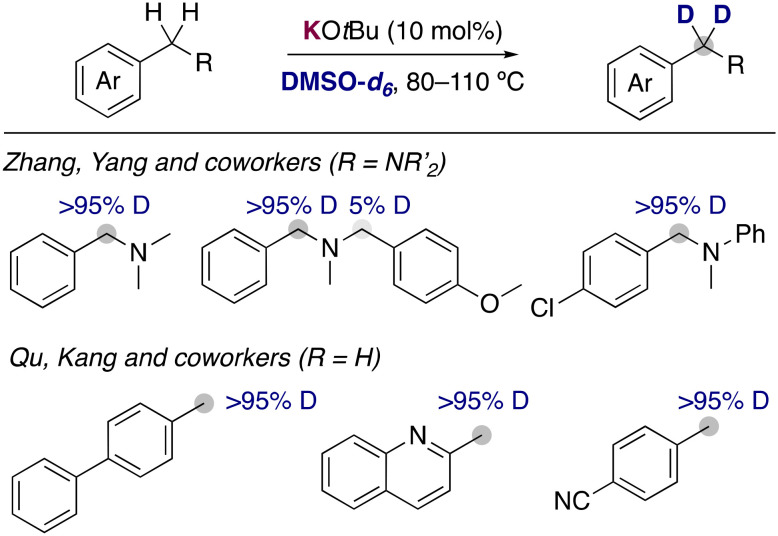
KO*t*Bu catalysed deuteration of benzylic positions.

With the aim of carrying out the deuterium incorporation in milder conditions, the use of easily accessible DMSO-*d*_6_ has become a good alternative for the deuterium incorporation. It is known that different solvents are able to stabilize in a different way the organic anion resulting from the metalation, causing a different experimental p*K*_a_ for the same substrate depending on the solvent used.^10^ These different properties play a key role in the deuteration *via* HIE using alkali-metal bases, allowing for reactivities in DMSO that are not possible with D_2_O under the same reaction conditions. The first report of the use of alkali-metal bases with DMSO-*d*_6_ was in 1970, when Leitch and co-workers reported the use of NaH for the deuteration of toluene and other methyl substituted aromatics.^11^ High temperatures (up to 170 °C) and multiple cycles were necessary to achieve good isotopic incorporation. Despite this early report, the development of new methodologies using this deuterated solvent remained scarce until 2015, when a new example of alkali-metal base catalysed deuteration was reported. Zhang, Yan and co-workers disclosed the use of catalytic amounts of KO*t*Bu in DMSO-*d*_6_ to incorporate selectively deuterium atoms into the benzylic position of benzylic amines (Fig. 3).^12^ A few years later, the group of Qu and Kang reported that similar condition at slightly higher temperatures were able to deuterate the benzylic position of a different array of arenes, where they could tolerate the presence of heterocycles or nitrile groups that are often not compatible with the presence of polar organometallic reagents.^13^ Key mechanistic experiments with a radical clock and performing the reaction in presence of TEMPO showed that the involvement of radical pathways in this transformation is unlikely, proposing an anionic pathway for the HIE reaction *via* reversible protonation/deprotonation of the benzylic position and the solvent by KO*t*Bu.

**Fig. 4:**
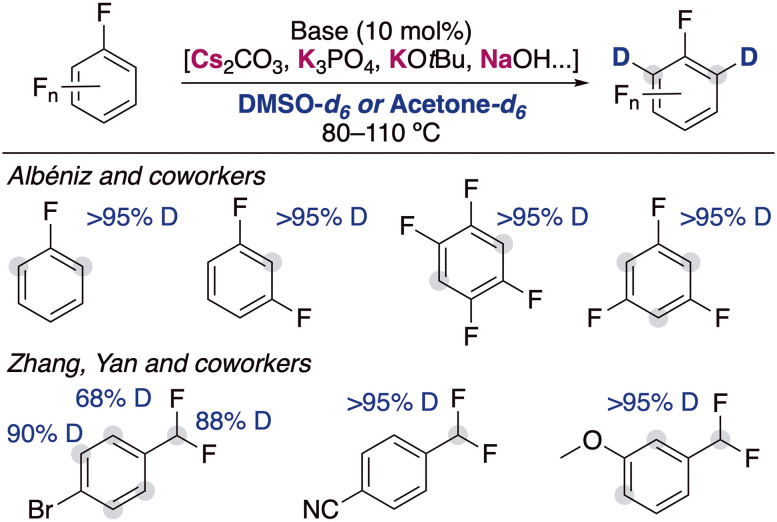
Deuteration of fluoroarenes with alkali-metal bases.

The inclusion of fluorine substituents in aromatic groups enhances the acidity of hydrogens located at their *ortho*-positions,^14^ making their metalation, in principle, more favoured. However, the main limitation of this approach is the thermal fragility of the generated metalated intermediates which can undergo decomposition *via* unwanted side reactions (*i.e.*, benzyne formation, autometalation or cascade processes).^15^ The use of mild bases in catalytic amounts circumvents this problem and allow the incorporation of deuterium in the *ortho*-position of different fluoroarenes (Fig. 4). Albéniz and co-workers described the used of Cs_2_CO_3_ and K_3_PO_4_ as efficient catalysts at relatively high temperatures in DMSO.^16^ They reported the possibility of scaling these reactions up (5 mmol) and found a correlation between the calculated acidities by DFT calculations and the experimental reactivity observed, which supported that the mechanism of the reaction involved the deprotonation of the fluoroarenes by the alkali-metal base. Zhang, Yan and co-workers reported recently that KO*t*Bu can be also employed for the deuteration of polyhalogenated arenes, observing deuterium incorporation in the position adjacent to the halogen substituents, as well as in the benzylic position of substituted α,α-difluorotoluenes.^17^ In this report the authors propose a similar mechanistic rational, showing a clear alkali-metal effect where the use of NaO*t*Bu resulted in lower deuterium incorporations and with LiO*t*Bu no deuterium incorporation was observed at all. Moreover, it is suggested that the presence of D_2_O can decrease the basicity of the medium, resulting in a lower isotopic incorporation but in some cases also in better selectivity of deuterium incorporation into the benzylic position.

**Fig. 5:**
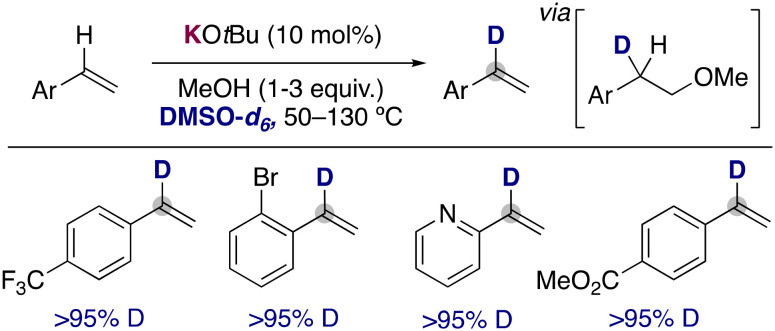
α-Selective deuteration of styrene derivatives.

In 2019, the group of Bandar reported that the use of catalytic KO*t*Bu in the presence of MeOH using DMSO-*d*_6_ as solvent could promote the deuterium incorporation into the α-position of styrene derivatives. They proposed a key role for methanol in this transformation, where it allows the transient deutero-alkoxylation of the double bonds, enabling the selective incorporation of the deuterium atoms in the styrene moiety (Fig. 5).^18^ In addition, the analysis of the reaction profile with different amounts of the alcohol also showed a critical role of MeOH in enabling the deuteration over competing side reactions, such as polymerization. The procedure can be applied to a wide range of styrene derivatives, tolerating ester groups, bromides or even heterocycles. It is also remarkable that under these conditions, the metalation of the arene ring or the polymerization of the alkenes is not observed, allowing the synthesis of α-deuterated styrenes that could be employed in the formation of deuterated stereodefined compounds by further functionalizing the double bond (*e.g.* dihydroxylation, cyclopropanation and hydroamination). Related deuteroalkoxylation and deuteroamination of alkynes have also been reported with KO*t*Bu in DMSO-*d*_6_,^19^ where the deuteration of the acidic terminal position of the alkyne^20^ is combined with the addition across the triple bond of the alkoxide to prepare the corresponding deuterated vinyl ethers or amines.

**Fig. 6:**
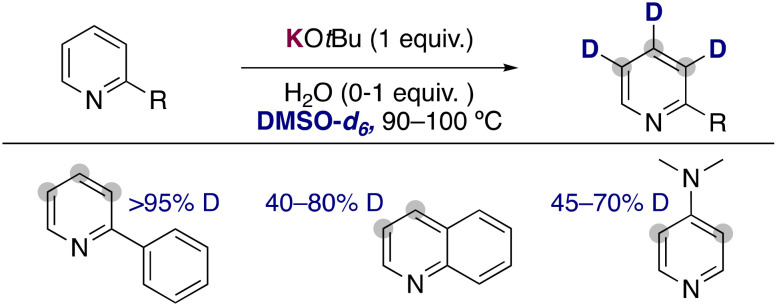
Deuteration of pyridine with KO*t*Bu in DMSO-*d*_6_.

Expanding the substrate scope of these approaches, the groups of Beller^21^ and Gao^22^ have independently reported the use of stoichiometric amounts of KO*t*Bu in DMSO-*d*_6_ as an efficient combination to incorporate deuterium atoms into the aromatic ring of pyridines and related heterocycles (Fig. 6). In contrast with the HIE catalysed by transition metals, where the pyridine ring directs the metalation and dictates the site of exchange, in these examples the deuterium atoms are incorporated into the 2-, 3- and 4-position of the pyridine ring. They could see that as previously discussed with the work of Zhang, in some cases the addition of small amounts of water into the reaction media had a beneficial effect in the deuterium incorporation or the isolated yields obtained. Moreover, the group of Beller performed some DFT calculations to rationalize the regioselectivity observed in the isotope exchange. The more reactive position matched with the more stable calculated carbanions, suggesting that a polar pathway is also operative in these processes, where the potassium alkoxide is able to partially deprotonate both the pyridine and the deuterated solvent and allow the transfer of deuterium into the aromatic ring. This is also in agreement with the pronounced alkali-metal effect observed in this transformation, where the use of less basic NaO*t*Bu and LiO*t*Bu lead to no deuterium incorporation at all.

**Fig. 7:**
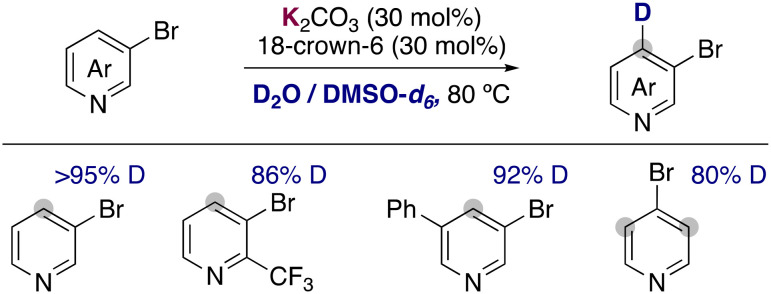
Deuteration of pyridine with K_2_CO_3_/18-crown-6 in D_2_O/DMSO-*d*_6_.

Recently, the group of Zhang has described the use of the milder base potassium carbonate in combination with 18-crown-6 to promote the deuteration of more fragile 3-bromopyridines and related heterocycles using a mixture of D_2_O and DMSO-*d*_6_ as solvent (Fig. 7).^23^ Remarkably the hydrogen isotope exchange is observed exclusively in the 4-position of the pyridine ring, in the *ortho*-position to the bromo group. The tolerance of a bromine allowed the further functionalization of the deuterated compounds *via* palladium catalysed cross-coupling, forming C–C bonds to access biaryls, alkynes, alkenes or esters. Key for the success of this reaction were the low basicity of potassium carbonate, which could be enhanced with the addition of 18-crown-6. The use of more basic KO*t*Bu lead to the obtention of debrominated products and the reaction in the absence of the crown ether resulted in low deuterium incorporation. In a similar way to the examples described before, key mechanistic experiments with TEMPO and kinetic experiments supported a carbanionic pathway for the deuteration of bromopyridines.

### Using less reactive C_6_D_6_ or D_2_/T_2_ for base catalysed HIE

**Fig. 8:**
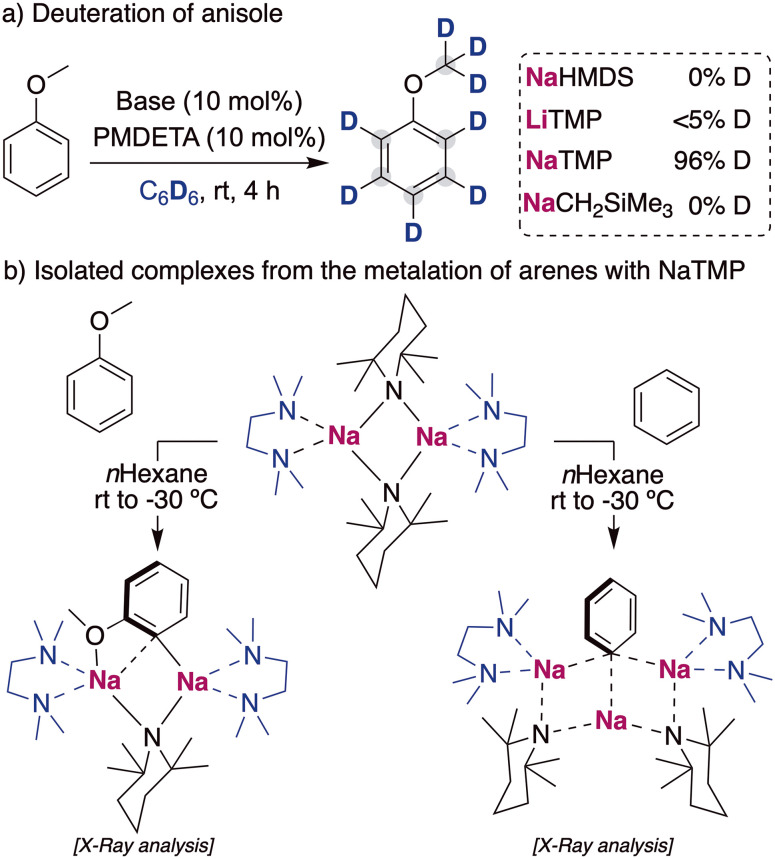
Influences of alkali-metal bases on the deuteration of anisole with C_6_D_6_ and isolation of metalated species in the solid state.

When considering other deuterium sources for HIE processes, iridium and other transition metal catalysts have used deuterated benzene or D_2_/T_2_ extensively. These compounds are less reactive deuterium sources for base mediated HIE, due to the higher p*K*_a_ of the C–H bond in benzene (∼43)^24^ or the H–H in H_2_ (∼49).^25^ Therefore, the use of these compounds as deuterium sources requires the use of more potent alkali-metal bases that allow the reversible deprotonation of said molecules to allow the transfer of the deuterium atoms to the desired target products. While *a priori* using stronger metalating reagents can be perceived as a limitation in terms of functional group tolerance and substrate scope, it can also open new opportunities to allow HIE of non-activated molecules containing hydrogen atoms with reduced acidity, which are out of reach with the alkali-metal base methods discussed above under mild reactions conditions. In this regard, the Hevia group have investigated the use of different alkali-metal basic amides for the deuteration of anisole in deuterated benzene. These studies have revealed that the use of polydentate amines, such as PMDETA (*N*,*N*,*N*′,*N*′′,*N*′′-pentamethyldiethylenetriamine), were key for increasing the solubility and promoting the deaggregation of the alkali-metal bases, enhancing their kinetic basicity. When different bases were tested in catalytic amounts, the less basic NaHMDS (HMDS = 1,1,3,3,3-hexamethyldisilazide) and LiTMP (TMP = 2,2,6,6-tetramethylpiperidide) were not able to catalyse the incorporation of deuterium into the aromatic compound. However, when the sodium amide NaTMP was used, full deuteration of every position of the molecule was observed, including the –OCH_3_ group (Fig. 8a).^26^ The use of an even more basic sodium alkyl NaCH_2_SiMe_3_ resulted in no deuterium incorporation, with the observation of just the sodiated arene, evidencing the requirement of a reversible metalation facilitated by the sodium amide to observe deuterium incorporation. Mechanistic investigations revealed that under stoichiometric conditions using C_6_H_6_ or hexane as a solvent the starting materials are in equilibrium with the relevant sodium aryl and the amine TMP(H).^27^ Further evidence on the metalating ability of NaTMP to deprotonate anisole and benzene was obtained by the isolation and structural identification of the organometallic species involved in these reactions (Fig. 8b) which can be envisaged as co-complexes between the metalation products NaAr (Ar = C_6_H_4_–OMe, C_6_H_5_) and the sodium amide NaTMP·L (L = TMEDA, *N*,*N*,*N*′,*N*′-tetramethylethylenediamine). Two different structural motifs were uncovered depending on the substrate employed, containing either two or three Na centers.^26,27^

**Fig. 9:**
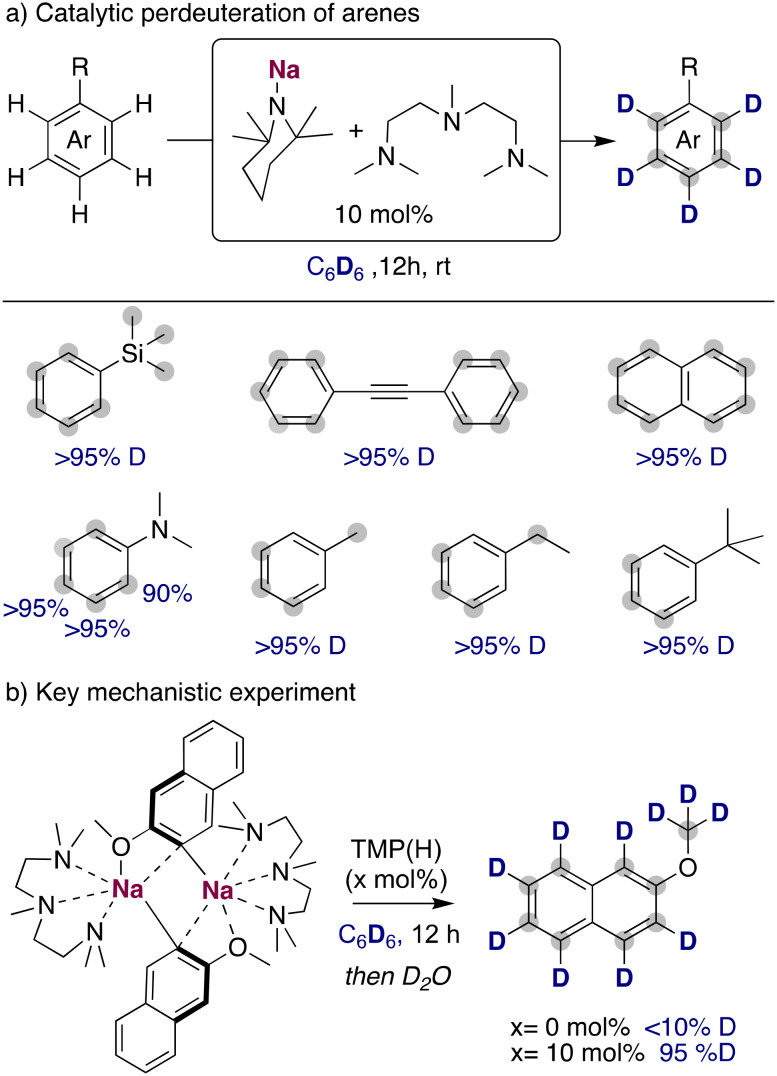
Deuteration of unactivated arenes with NaTMP/PMDETA in C_6_D_6_.

The use of the NaTMP/PMDETA combination for the perdeuteration of aromatic molecules could be extended to other less reactive substrates, such as phenyl trimethyl silane, diphenyl acetylene or naphthalene (Fig. 9a), achieving high deuterium incorporations with relatively mild conditions (room temperature and a reaction time of twelve hours). Complete deuteration was observed as well in the benzylic position of alkyl-substituted arenes, where a slower deuterium incorporation was observed in the *otho* position to the alkyl substituents, probably due to steric reasons that prevented the efficient metalation of these positions with the bulky sodium amide. One of the main limitations of this methodology remains to be the functional group tolerance, where more sensitive groups like ketones, esters or nitriles are not compatible with the organosodium intermediates formed in solution. To further explore the role of the TMP(H) in this catalytic process, sodiated 2-methoxynaphthalene was prepared, isolated as a pure compound and then dissolved in C_6_D_6_. In the absence of TMP(H), no deuterium incorporation was observed. However, upon addition of a catalytic amount of TMP(H) (10 mol%), a high degree of deuterium incorporation was obtained, proving the key role of the TMP base for an efficient HIE process (Fig. 9b). With this information, it is proposed that NaTMP is able to deprotonate the substrates and the deuterated solvent, producing low amounts of TMP(H/D) that are key for the transfer of the deuterium atoms from the solvent into the products. Expanding further the synthetic utility of this approach, this sodium mediated HIE process can be upscaled and combined with a sodium-mediated borylation step to afford deuterated boronic acids in high yields. These are highly valuable intermediates to access new deuterated scaffolds in a modular manner.

The use of isotopically labelled D_2_ and T_2_ in alkali-metal base mediated HIE has remained a challenge, due to the low reactivity of this gas with bases. The use of said gases is particularly important in the case of tritium labelling, since the main sources of this radioactive isotope are diluted T_2_O (in its pure form is highly toxic and unstable due to autoradiolysis) and T_2_ gas. The first is not usually useful to incorporate this isotope into C–H bonds *via* HIE, so the use of T_2_ is the stablished method for a practically and convenient tritiation, which also allows the use of the well-stablished manifolds used with transition-metal catalysed protocols.

In 2018, the group of Stephan described the ability of alkali-metal hydrides, phosphides and amides to reversibly activate dihydrogen enabling both isotopic scrambling and catalytic hydrogenation. These studies showed that low but significant levels of deuterium incorporation could be achieved into the benzylic position of toluene. Insightful DFT investigations showed that the reactions take place *via* the interaction of H_2_ with the alkali-metal (which acts as a Lewis acid) and the basic phosphide or amide group, in a mechanism which is reminiscent to that previously proposed for the activation of H_2_ by FLP systems.^28^ Two years later, Harder and co-workers also reported the ability of related alkaline-earth metal amides (Ca, Sr and Ba) to catalyse the HIE process between aromatic systems and H_2_.^29^ In this case they propose that the metal hydrides formed by reaction of the amides with H_2_ are the active intermediates, which undergo a nucleophilic attack into the aromatic ring, enabled by the soft Lewis acid–base interaction of the alkaline-earth cation with the aromatic motive.

**Fig. 10:**
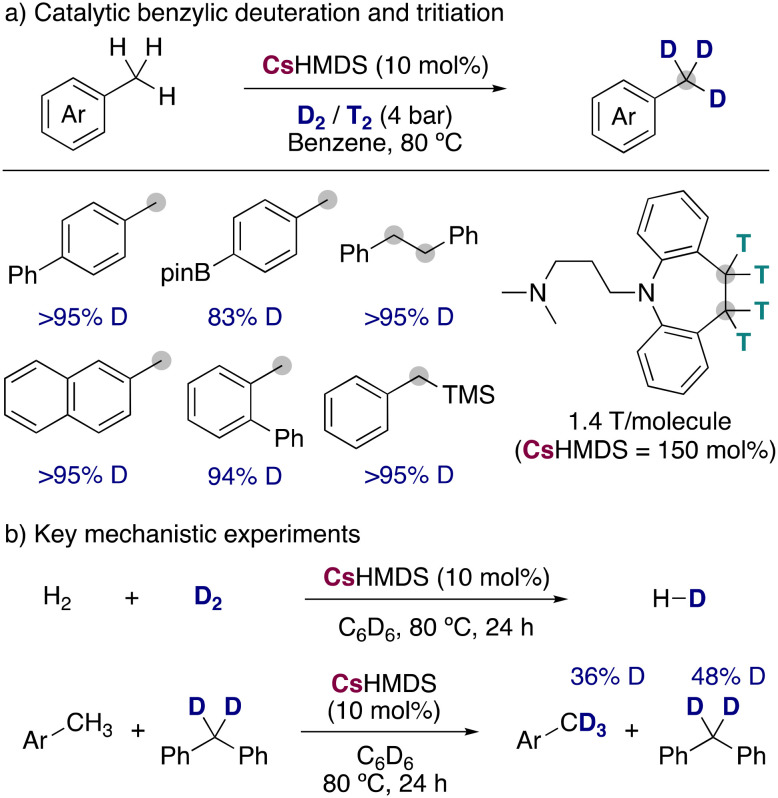
Deuteration and tritiation of benzylic positions with CsHMDS.

Expanding the synthetic scope and improving the isotopic incorporations, Yang, Guan and co-workers reported the use of CsHMDS in catalytic amounts for the incorporation of deuterium and tritium into benzylic positions, using low pressures of the corresponding labelled gases (Fig. 10a).^30^ Remarkably, the milder conditions allowed a better functional group tolerance but restricted the isotopic incorporation to the benzylic positions, not being observed any HIE in the less acidic C–H bonds of the aromatic ring. Different substituents and functional groups were tolerated, and it was even applied to the radiochemical labelling of active pharmaceutical ingredients, being this the first example of alkali-metal bases used for the tritiation of organic molecules. An important alkali-metal effect was also observed in this reaction, with LiHMDS and NaHMDS being not active at all, KHMDS leading to modest deuterium incorporations (∼40%) and RbHMDS giving similar results than CsHMDS. To shed light into the possible mechanism of the reaction, the authors performed some key mechanistic experiments (Fig. 10b) showing that CsHMDS under the catalytic conditions is able to activate both D_2_ and the benzylic C–H bonds. Further experiments with a radical clock could show that a radical pathway was most probably not occurring, proposing a polar mechanism in which the caesium amide is able to react with both the substrates and D_2_, allowing the incorporation of the deuterium into the benzylic position of the arenes.

## Conclusions and outlook

The use of alkali-metal bases has become in recent years a viable alternative to transition metals for hydrogen isotopic exchange in organic molecules. The use of different solvents, deuterium sources or additives, as well as the nature of the alkali-metal and the counteranion have allowed to modulate the reactivity of these reagents, enabling the deuterium incorporation into fragile heterocycles or very unreactive aromatic compounds such as naphthalene or toluene. This variation of the metalating power of the bases has facilitated the use of different deuterium sources as well, moving from the supercritical D_2_O to DMSO, benzene or D_2_. This last example could also be translated to its tritium analogue, showing that alkali-metal amides are also competent in the radiolabelling of pharmaceuticals with this isotope, with the benefit of reducing the radioactive waste when using T_2_ compared with T_2_O.

Despite all these advances, the detailed study of the reaction mechanism is still lacking in some of these transformations. DFT calculations and key mechanistic experiments have been performed in a few examples,^13,16,18,21,23,27,28,30^ but in general the specific mechanistic details of these processes are still poorly understood. We predict that further developments on the use of alkali-metal bases for HIE processes will keep appearing, which in combination with a deeper mechanistic understanding will bring the use of alkali-metal bases as an excellent pathway for the incorporation of deuterium and tritium into organic molecules, with the benefits of using widely available and cheap alkali-metal reagents. Improving the regioselectivity to achieve the isotopic exchange in the desired C–H bonds and preserving the chemoselectivity will be key to bring the alkali-metal catalysed HIE to the forefront of the field.

## Author contributions

A. Tortajada and E. Hevia designed and wrote the publication.

## Conflicts of interest

There are no conflicts to declare.

## Supplementary Material
